# Virginia State Board of Veterinary Medical Examiners

**Published:** 1901-11

**Authors:** 


					﻿VIRGINIA STATE BOARD OF VETERINARY MEDICAL
EXAMINERS.
Dr. H. Bannister, of Roanoke, Secretary of the Board,
reports no changes in the law during the past year. The same
rules and regulations of the Board are in force.
The following veterinarians were given the license of the Board
the past year: F. A. Smith, V.S.; D. Eugene Block, V.S.,
graduate of Ontario Veterinary College, class of 1892; Charles
H. Epps, V.S., graduate of Ontario Veterinary College, class of
1901; and Thomas Fraser, V.S., graduate of Ontario Veterinary
College, class of 1901.
				

